# Wall motion assessment by feature tracking in pediatric patients with coronary anomalies undergoing dobutamine stress CMR

**DOI:** 10.3389/fcvm.2024.1380630

**Published:** 2024-06-11

**Authors:** Shagun Sachdeva, Silvana Molossi, Dana Reaves-O’Neal, Prakash Masand, Tam T. Doan

**Affiliations:** ^1^ Pediatric Cardiology, Baylor College of Medicine, Houston, TX, United States; ^2^ Pediatric Radiology, Baylor College of Medicine, Houston, TX, United States

**Keywords:** coronary anomalies, stress cardiac MRI, strain imaging, dobutamine, wall motion abnormalities (WMA)

## Abstract

**Background:**

Left ventricular (LV) wall motion assessment is an important adjunct in addition to perfusion defects in assessing ischemic changes. This study aims to investigate the feasibility and utility of performing feature tracking (FT) in pediatric patients with coronary anomalies undergoing dobutamine stress CMR to assess wall motion abnormalities (WMA) and perfusion defects.

**Method:**

This is a retrospective study where 10 patients with an inducible first-pass perfusion (FPP) defect and 10 without were selected. Global LV circumferential strain/strain rate (GCS/GCSR) was measured at rest and at peak stress (systole and diastole) using a commercially available feature tracking software. Peak GCS and GCSR were compared to indexed wall motion score (WMSI) between groups with and without FPP defect and in subjects with and without WMA.

**Results:**

The median age of patients was 13.5 years (Q1, 11 years; Q3, 15 years). Five subjects had qualitatively WMA at peak stress. A moderate correlation of GCS with WMSI at peak stress (0.48, *p* = 0.026) and a significant difference between GCS at rest and stress in patients with no inducible WMA (*p* = 0.007) were seen. No significant difference was noted in GCS between rest and stress in patients with WMA (*p* = 0.13). There was a larger absolute GCS/GCSR at peak stress in subjects with no inducible FPP defect or WMA.

**Conclusion:**

Smaller absolute GCS and a lack of significant change in GCS at peak stress in those with inducible WMA or perfusion defect are suggestive of compromised LV deformation in subjects with inducible WMA. Given these findings, GCS derived from CMR-FT may be used to objectively assess WMA in pediatric patients undergoing stress CMR.

## Graphical Abstract

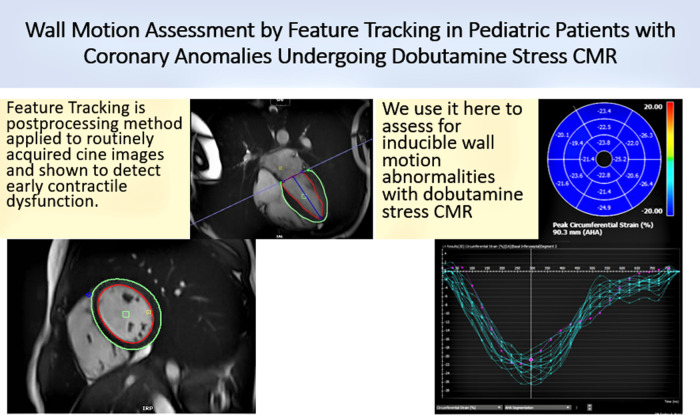

## Introduction

Coronary artery anomalies (CA) are an assorted group of congenital heart diseases with variable pathophysiology, clinical presentation, and implications. Of all the types, the anomalous aortic origin of a coronary artery (AAOCA) is identified as the one with the highest risk of sudden cardiac death (SCD) in young athletes (1, 2). The true prevalence of AAOCA in the general population remains unknown, as studies have focused primarily on symptomatic patients. The estimated frequency of anomalous aortic origin of the left coronary artery (AAOLCA) is 0.03%–0.15% while that of anomalous aortic origin of the right coronary artery (AAORCA) is 0.28%–0.92% (3). AAOCA is known to be the second leading cause of SCD in young athletes estimated to be responsible for 15%–20% of sudden deaths in this population (1, 2, 4, 5).

**Figure 1:**
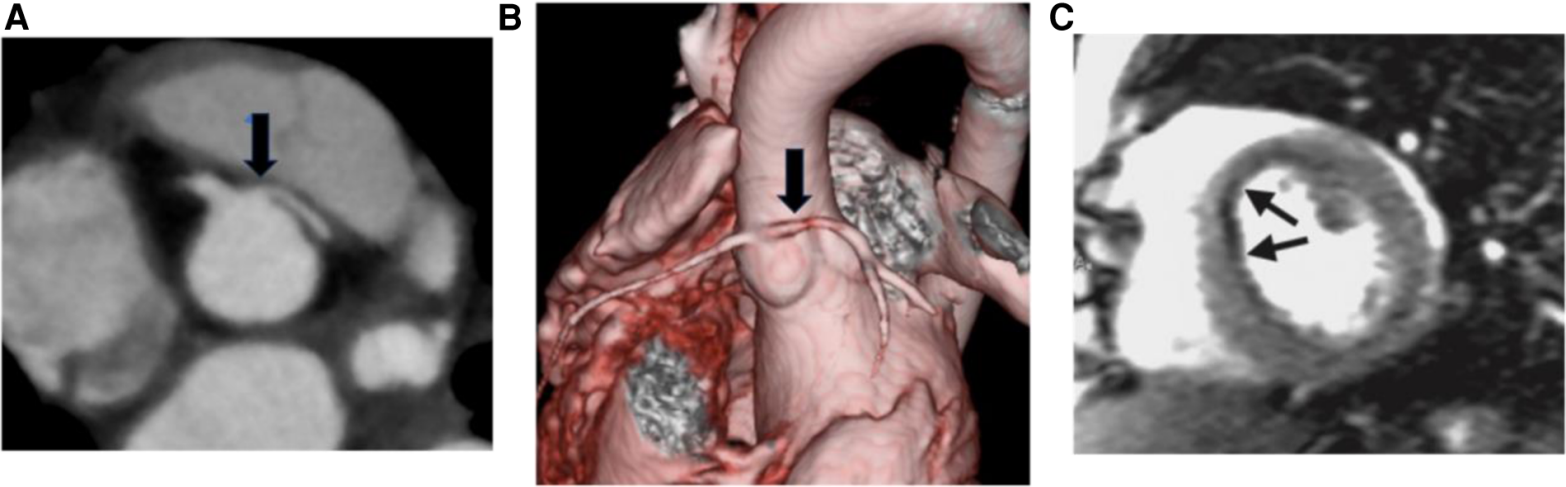
(**A**) Axial CTA image showing the anomalous aortic origin of the left coronary artery (L-AAOCA) (**B**) 3D reconstruction depicting the L-AAOCA. (**C**) Dobutamine stress cardiac MRI perfusion sequence showing a subendocardial perfusion defect in the anterior interventricular septum.

**Figure 2:**
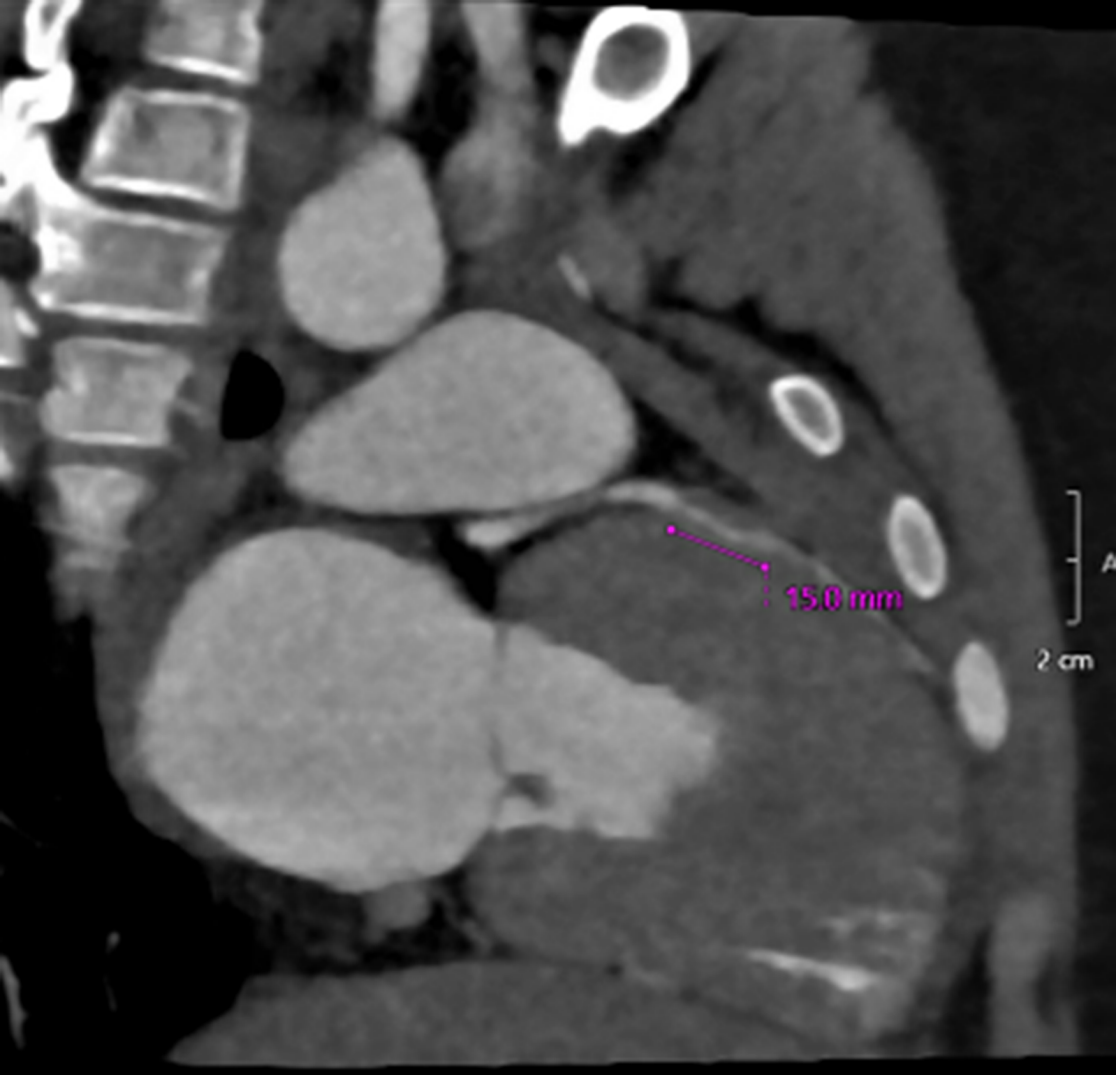
CTA image showing a left anterior descending coronary artery myocardial bridge.

Cardiovascular magnetic resonance with dobutamine stress (DSCMR) is increasingly being utilized in the functional assessment of CA, including AAOCA and myocardial bridges (MB), given that it is non-invasive and utilizes no radiation (6) (Figures 1, 2). It not only provides volumetry data and ejection fraction but also identifies the presence of perfusion defects at rest and with pharmacological stress (6). It has been deemed safe and feasible in the pediatric population (6). The assessment for left ventricular (LV) wall motion abnormalities (WMA) is an important adjunct in assessing ischemic changes (7). It is conventionally performed by subjective visual examination, which is associated with considerable interobserver variability (8). The wall motion score index (WMSI) reflects the magnitude of myocardial damage and the total extent of WMA (8).

CMR myocardial feature tracking (CMR-FT) is utilized for circumferential and longitudinal myocardial mechanics (9, 10). This tool has been reported to be used in adults to predict functional recovery at rest in patients with coronary artery disease (11). Deformation imaging has the potential as a tool to detect early/subclinical changes in LV function in a variety of congenital and acquired heart diseases (12–15) and as a surrogate measure of outcome in therapeutic trials (16). However, there is limited data in the pediatric population particularly with AAOCA regarding utilization of this tool (14, 17, 18).

In this study, we evaluated the feasibility and utility of performing CMR-FT in pediatric patients undergoing stress CMR to assess WMA and perfusion defects in coronary anomaly patients as part of risk stratification for management decision-making.

## Methods

Twenty patients with coronary anomalies who underwent a DSCMR as a part of their evaluation at the Coronary Artery Anomalies Program at Texas Children's Hospital were selected from our database and included in this study. Ten of these patients had an inducible first-pass perfusion (FPP) defect with dobutamine infusion and 10 did not. These two groups were matched for gender and type of anomaly.

### DSCMR

**Figure 3:**
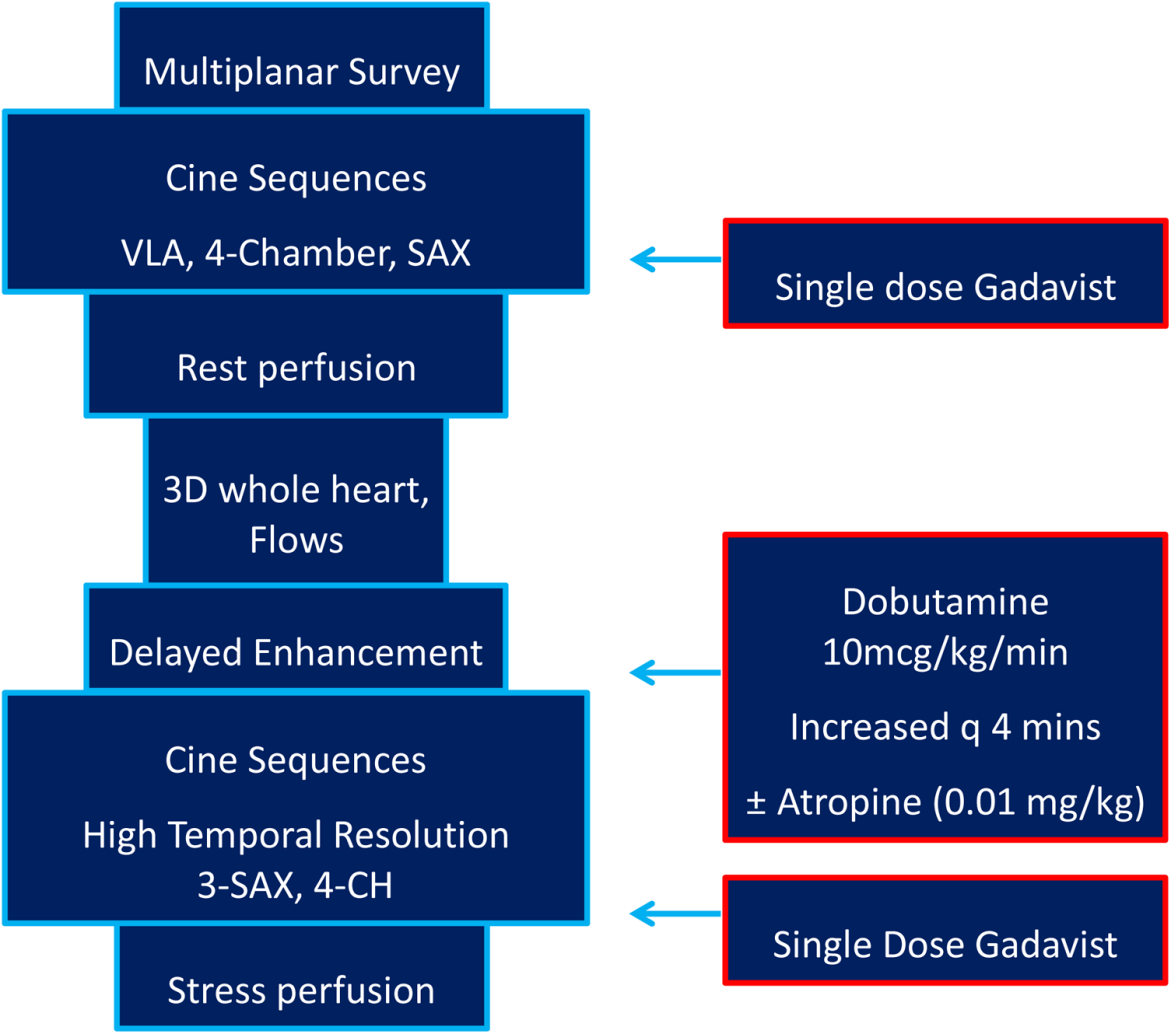
Institutional dobutamine stress cardiac MRI imaging protocol.

All the studies were performed on a 1.5 T Magnetom Aera (Siemens Healthineers, Erlangen, Germany) scanner. Initially, cine imaging with SSFP sequences was performed in ventricular long-axis, four-chamber, and short-axis planes. Myocardial perfusion was then assessed at rest with 0.1 mmol/kg of gadolinium-based contrast agent (Gadavist, Bayer HealthCare Pharmaceuticals, ON, Canada). Late gadolinium enhancement (LGE) sequence in short-axis and four-chamber orientation was performed around 5 min after contrast administration to assess myocardial viability. Dobutamine was started at 10 μg/kg/min and increased every 4 min to a maximum of 40 μg/kg/min. Additional atropine 0.01 mg/kg was given intravenously if the target heart rate (HR) was not attained. A HR of at least 150 or a rate pressure product of (RPP = HR × systolic blood pressure) ≥ 20 × 10^3^sbpm mmHg was used as a minimal target hemodynamic response (6, 19). Cine SSFP sequences in the short-axis (basal, mid, and apical slices) and four-chamber orientation were performed with breath-hold if possible at each dobutamine dose to assess for WMA. A repeat FPP sequence was performed with a second dose of gadolinium. We aimed for a temporal resolution of the cine sequences ≤40 msec/frame and spatial resolution of the FPP ≤2.2 × 2.2 mm. Our institutional imaging protocol is detailed in Figure 3.

### Indexed wall motion score

We assessed WMA using a four-point wall motion scoring system used to derive an indexed wall motion score (WMSI) according to the AHA 16-segment model (20, 21). Each segment was analyzed individually and scored based on its motion and systolic thickening. Each segment's function was confirmed in multiple views. Segments were scored as follows: normal or hyperkinesis = 1, hypokinesis = 2, akinesis = 3, and dyskinesis (or aneurysmatic) = 4 (22). WMSI was derived as the sum of all scores divided by the number of segments visualized into a single parameter (22). WMSI > 1 was taken as suggestive of wall motion abnormality (23).

### CMR-FT

**Figure 4:**
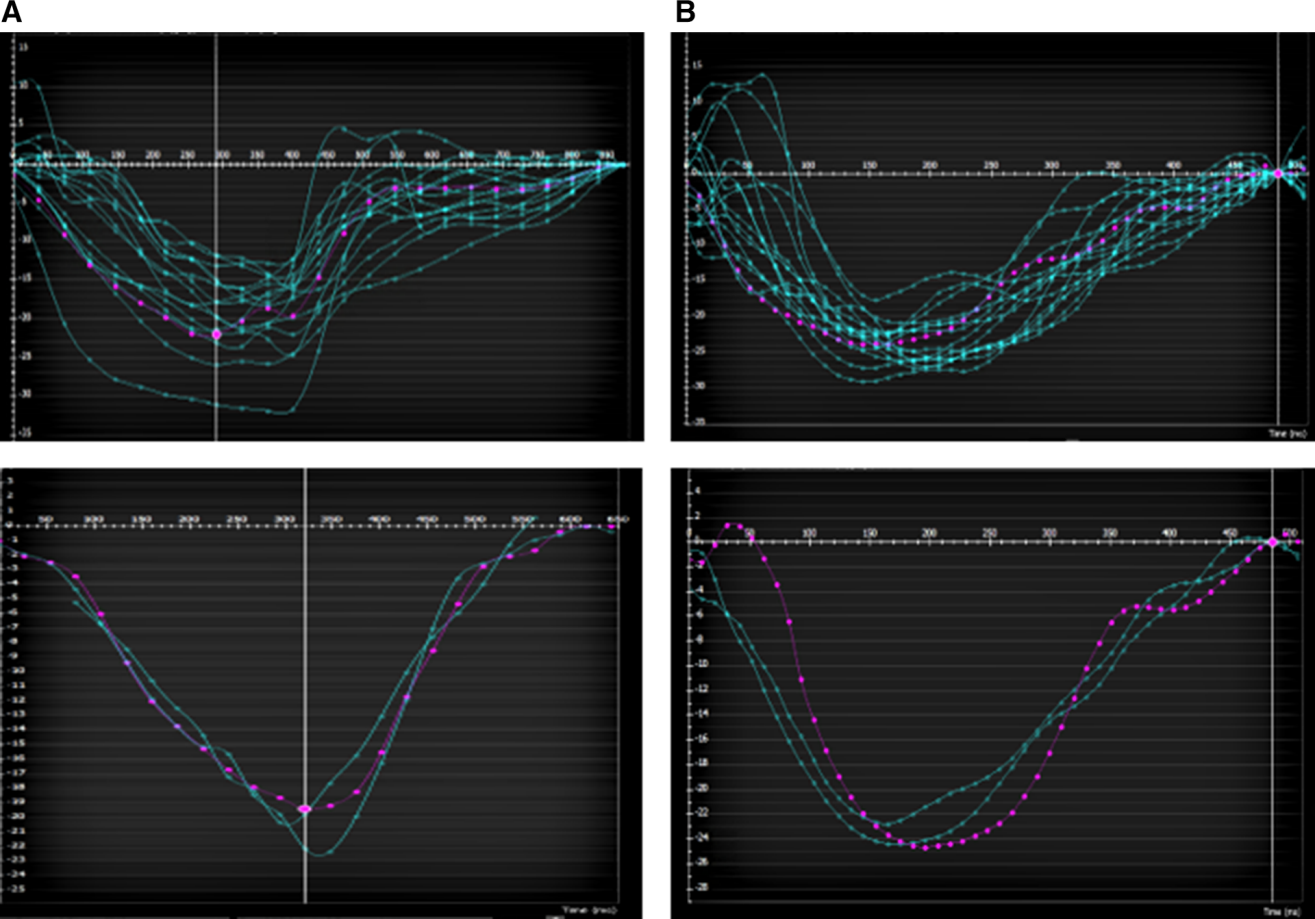
Example of a patient with LAD myocardial bridge with homogenous contraction (by AHA segments top and by apical/mid/basal slice bottom) (**A**) at rest (**B**) and at peak stress.

**Figure 5:**
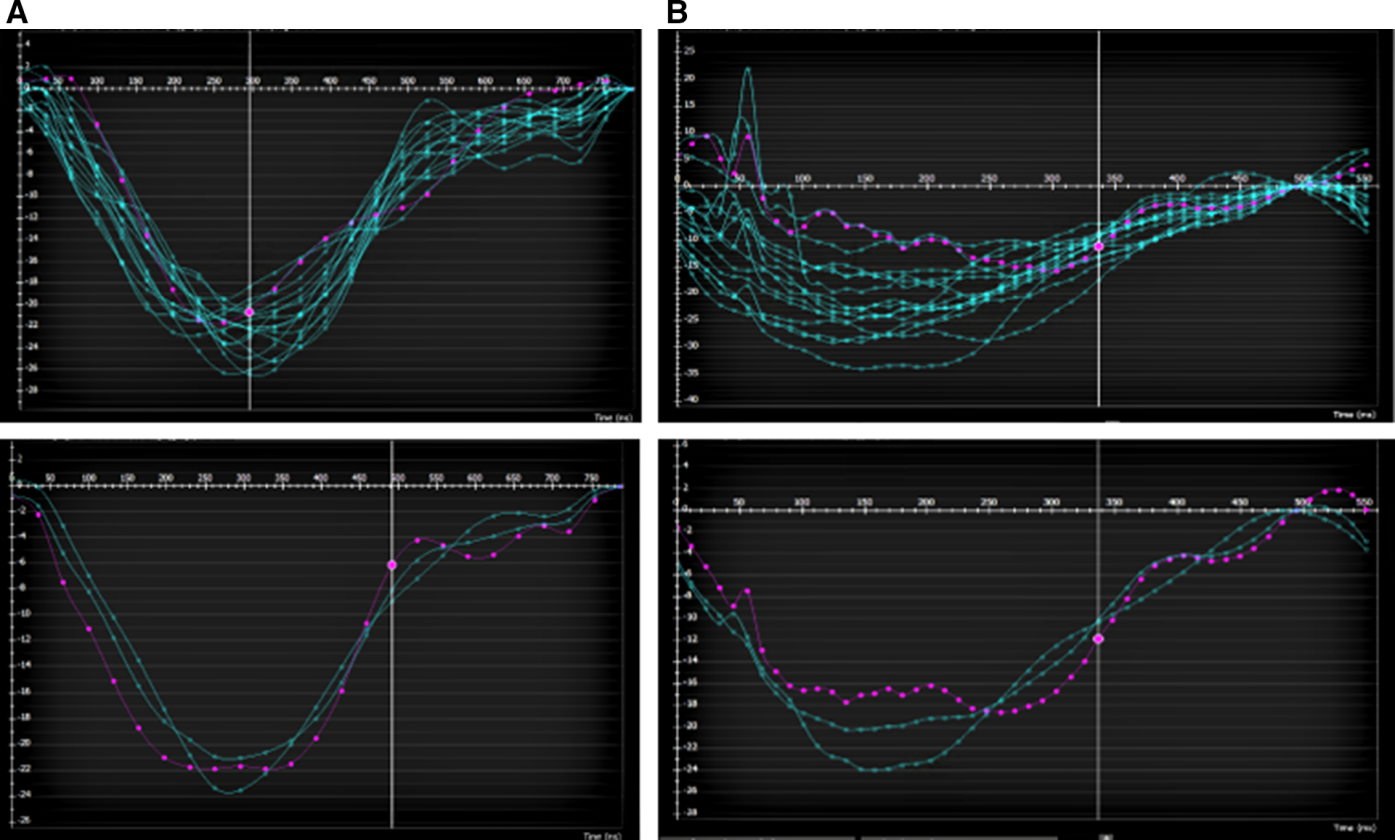
Example of a patient with LAD myocardial bridge with (**A**) homogenous contraction (by AHA segments top and by apical/mid/basal slice bottom) at rest (**B**) and heterogenous contraction (by AHA segments top and by apical/mid/basal slice bottom) at peak stress especially in the apical and basal slices.

Global LV circumferential strain/strain rate (GCS/GCSR) was measured following automated LV endo- and epicardial tracking at rest and at peak stress (in systole and diastole) using feature tracking software on CVI42. Rest GCS and GCSR were measured from representative basal, mid, and apical slices from the short-axis stack and using a four-chamber sequence for reference points. Stress GCS and GCSR were measured from the SSFP short-axis and four-chamber wall motion sequence. Since only one slice in the four-chamber view is acquired for stress wall motion assessment, change in longitudinal strain/strain rate was not studied. Endocardial contours were manually drawn in all analyzed slices by one skilled observer (SS). A second observer (TD) reanalyzed the images to assess interobserver variability. Examples of strain assessment given by AHA segments and slices are shown in Figures 4, 5.

### Statistics

The variables were reported as mean and standard deviation or median and interquartile ranges as appropriate. A Student’s *t*-test or Wilcoxon rank-sum test was used to compare the continuous variables, and chi-squared or Fisher’s exact test was used to compare the categorical variables of different groups. The Wilcoxon signed-rank test was used for repeat measures of hemodynamic changes and strain changes. Peak GCS and GCSR were compared to WMSI and compared between the two groups with and without inducible FPP defect and in subjects with and without WMA.

## Results

### Patient population

The mean age of the cohort at the time of the study was 13 years (range, 7–17 years). The diagnoses in 10 patients with inducible FPP defect included four anomalous aortic origins of the right coronary artery (R-AAOCA), two anomalous aortic origins of the left coronary artery (L-AAOCA), and for MB. Additionally, the diagnoses in the 10 patients without an inducible FPP defect included eight R-AAOCA, one L-AAOCA, and one MB. The stress medication used was dobutamine in all the subjects. Five of the 10 patients with a positive inducible perfusion defect had reported WMA by WMSI*.* There was a statistically significant difference in age between those with/without inducible perfusion abnormalities (Table 1).

**Table 1:** Patient demographics.

Parameter	With inducible perfusion defect (*n* = 10)	Without inducible perfusion defect (*n* = 10)	*p*-value
Median age, years (Q1, Q3)	15 (13,17)	11 (9,14)	**0**.**012**
Male gender (%)	9 (90%)	7 (70%)	0.364
Coronary anomaly			0.228
AAOLCA, *n* (%)	2 (20%)	1 (10%)	
AAORCA, *n* (%)	4 (40%)	8 (80%)	
MB, *n* (%)	4 (40%)	1 (10%)	
Median LVEF, % (Q1, Q3)	59 (57, 62)	60 (58, 63)	0.254
Intramural course, *N* (%) Median length, mm (Q1, Q3)	4 (40%) 6.0 (5.7, 6.1)	8 (80%) 6.0 (4.7, 6.3)	0.170 0.927
High (STJ or higher) takeoff, *N* (%)	2 (20%)	5 (50%)	0.350
Slitlike ostium, *N* (%)	2 (20%)	7 (70%)	0.370

AAOLCA, anomalous aortic origin of the left coronary artery; AAORCA, anomalous aortic origin of the right coronary artery; MB, myocardial bridge; LVEF, left ventricular ejection fraction; STJ, sinotubular junction.

The Wilcoxon rank-sum test was used for the non-parametric variables, and Fisher’s exact test was used for the categorical variables.

Bold value indicate significant *p*-value <0.05.

The image quality was sufficient to perform strain analysis in all segments for all subjects. LV and RV volumes were within normal limits (Table 1). There was no evidence of LGE in any of the included patients. There were no side effects to dobutamine exposure. There was a significant (*p* < 0.05) increase in HR, mean blood pressure, and cardiac output between rest and stress in the two groups (Table 2).

**Table 2:** Hemodynamic parameters.

Parameter	With inducible perfusion defect	Without inducible perfusion defect	*p*-value
Resting hemodynamics			
HR bpm	75 (70–80)	82 (77–92)	**0**.**027**
SBP mmHg	116 (110–120)	109 (102–114)	0.211
DBP mmHg	71 (56–74)	55 (47–67)	0.284
Stress hemodynamics			
HR bpm	152 (140–160)	149 (135–162)	0.703
SBP mmHg	167 (166–170)	149 (138–167)	0.169
DBP mmHg	77 (72–95)	70 (62–90)	0.464
RPP	25,272 (25,050–26,163)	22,370 (20,700–22,940)	0.273

DBP, diastolic blood pressure; HR, heart rate; SBP, systolic blood pressure.

The Wilcoxon rank-sum test was used for the non-parametric variables.

Bold value indicate significant *p*-value <0.05.

### Interobserver variability

Intraclass correlation coefficient analyses were performed between two readers and six subjects. There was very good interobserver agreement (*k* = 0.85) including GCS calculated at rest and stress.

### Assessment of strain

There was a moderate correlation of GCS with WMSI at peak stress (0.48, *p* = 0.026).

A significant difference was present between GCS at rest and stress in patients with no inducible WMA (*p* = 0.007) and also in GCS at rest and stress in patients with (*p* = 0.016) and without (*p* = 0.005) inducible FPP defect. However, the numerical change in value was higher in patients without inducible FPP defect.

A significant difference was again present when analyzing GCSR at rest and stress in patients with (*p* = 0.043) and without (*p* = 0.007) inducible WMA, and the numerical change in value was higher in patients without inducible WMA. In addition, a significant difference was demonstrated in GCSR at rest and stress in patients with (*p* = 0.005) and without (*p* = 0.005) inducible FPP defect; however, the numerical change in value was slightly higher in patients without inducible FPP defect (Table 3).

**Table 3:** Comparison of global GCS at rest and stress in patients with and without wall motion abnormality and with and without inducible perfusion defect.

	Rest: median (Q1, Q3)	Stress: median (Q1, Q3)	*p*-value	Total
Global GCS	−20.5 (−21.6, −19.1)	−24.9 (−27.4, −21.9)	**0**.**0001**	*N* = 20
WMA				
Inducible WMA	−20.6 (−21.5, −18.4)	−23.6 (−26.7, −20.5)	0.13	*N* = 5
No WMA	−20.4 (−22.4, −19.3)	−25.3 (−28.2, −22)	**0**.**007**	*N* = 15
FPP				
Inducible FPP defect	−20.8 (−21.5, −19.3)	−23.0 (−26.7, −21)	**0**.**016**	*N* = 10
No FPP defect	−20.0 (−23.5, −18.9)	−26.2 (−28.3, −22.4)	**0**.**005**	*N* = 10
Global GCSR	−1.16 (−1.31, −1.01)	−3.20 (−4.10, −2.40)	**0**.**0001**	*N* = 20
WMA				
Inducible WMA	−1.18 (−1.20, −1.07)	−2.58 (−3.90; −2.41)	**0**.**043**	*N* = 5
No WMA	−1.15 (−1.33, −0.96)	−3.30 (−4.30, −2.40)	**0**.**0007**	*N* = 15
FPP				
Inducible FPP defect	−1.14 (−1.21, −1.07	−3.20 (−4.30, −2.41)	**0**.**005**	*N* = 10
No FPP	−1.22 (−1.33, −0.96)	−3.25 (−3.50, −2.40)	**0**.**005**	*N* = 10

GCS, global circumferential strain; GCSR, global circumferential strain rate; FPP, first-pass perfusion; WMA, wall motion abnormality.

The Wilcoxon signed-rank test was used for the repeated measures.

Bold values indicate significant *p*-values <0.05.

No significant difference was seen between GCS/GCSR at rest and stress in patients with or without WMA. However, the numerical value of GCS/GCSR with stress was higher in patients without WMA. No significant difference was also appreciated between GCS/GCSR at rest and stress in patients with or without inducible FPP defect. Nevertheless, the numerical value of GCS at stress was higher in patients without inducible FPP defect (Table 3).

When comparing the GCS and GCSR in patients with and without WMA, there was a significant difference only noted at the base (*p* = 0.043). However, the numerical value of GCS/GCSR was higher in patients without WMA (Table 4). Similar findings were noted in patients with and without an inducible FPP defect (Table 5).

**Table 4:** Comparison of global GCS and GCSR in patients with and without wall motion abnormality.

	WMA+: median (Q1, Q3)	WMA−: median (Q1, Q3)	*p*-value	Total
GCS rest	−20.6 (−21.5, −18.4)	−20.4 (−22.4, −19.3)	0.51	*N* = 20
GCS stress	−23.6 (−26.7, −20.5)	−25.3 (−28.2, −22)	0.17	
GCS %change	22.7 (14.0, 23.0)	18.3 (7.7, 33.3)	0.76	
GCSR rest	−1.18 (−1.20, −1.07)	−1.15 (−1.33, −0.96)	0.86	
GCSR stress	−2.58 (−3.90, −2.41)	−3.30 (−4.30, −2.40)	0.63	
GCSR %change	125 (107, 230)	163 (130, 290)	0.40	

The Wilcoxon rank-sum test was used for the non-parametric variables.

**Table 5:** Comparison of global GCS and GCSR in patients with and without inducible perfusion defect.

	Induced hypoperfusion+: median (Q1, Q3)	No hypoperfusion−: median (Q1, Q3)	*p*-value
GCS rest	−20.8 (−21.5, −19.3)	−20.0 (−23.5, −18.9)	0.87
GCS stress	−23.0 (−26.7, −21)	−26.2 (−28.3, −22.4)	0.12
GCS %change	19.4 (9.7, 23.2)	18.3 (15.9, 33.3)	0.65
GCSR rest	−1.14 (−1.21, −1.07	−1.22 (−1.33, −0.96)	0.57
GCSR stress	−3.20 (−4.30, −2.41)	−3.25 (−3.50, −2.40)	0.93
GCSR %change	178 (107, 275)	143 (130, 208)	0.82

The Wilcoxon rank-sum test was used for the non-parametric variables.

In addition to the altered numerical value of GCS, the pattern of contraction was altered as well. There was a more homogenous contraction noted at rest and stress in patients without WMA and a heterogenous pattern with altered GCS in affected segments/slices with stress in patients with inducible WMA (Figures 4, 5).

## Discussion

To the best of our knowledge, this is one of the first studies to evaluate the use of CMR-FT in assessing for WMA in pediatric patients with AAOCA and comparing it in patients with and without perfusion defects [unlike the other recent study (17)]. We found that in subjects without WMA or inducible perfusion defect, there was a larger absolute GCS, along with a lack of significant change in the GCS at peak stress in subjects with inducible WMA, suggesting subtle compromised LV deformation in subjects with WMA and inducible perfusion defect. Importantly, we demonstrated that CMR-FT is feasible and reproducible even when the temporal resolution is limited by a fast HR in a DSCMR.

LV wall motion assessment is an important adjunct to perfusion imaging in assessing ischemic changes (7). The prognostic value of WMSI has been investigated in patients with acute STEMI and NSTEMI myocardial infarction as well as post-CABG, suggesting it provides incremental information in predicting infarct size and mortality (8, 21, 24, 25). However, there has been an incremental value to WMSI with strain/deformation imaging.

Myocardial deformation imaging has been shown to detect early contractile dysfunction in a number of cardiovascular diseases. This measures the degree of deformation of a myocardial segment from its initial length to its maximum length and is expressed as a percentage (10). Feature tracking technology is a post-processing method that can be applied to routinely acquired cine CMR images. It is based on identifying features in the image and tracking them in the successive images of the sequence (26). This way, the displacement of myocardial segments can be measured. Deformation imaging has the potential as a tool to detect early/subclinical changes in LV function in a variety of congenital and acquired heart diseases (12–15) and as a surrogate measure of outcome in therapeutic trials (16). This seems particularly desirable in patients with AAOCA where risk stratification of those patients at risk remains a challenge (27). Deformation imaging has previously been applied to dobutamine stress echocardiography, and it was shown to offer prognostic information that is independent and incremental to the standard WMSI (9). A study on healthy adult volunteers undergoing DSCMR assessed the feasibility of performing CMR-FT (11), but no studies have been performed on pediatric patients undergoing DSCMR and utilizing CMR-FT to assess for WMA and compare patients with and without perfusion abnormalities. Our study assesses CMR-FT in the pediatric population with coronary anomalies and compared abnormalities in strain in patients not only with and without WMA but also with and without perfusion defects.

In our study, we found that the performance of GCS and GCSR is feasible at rest and in stress wall motion sequences. Our study highlights some important features including the lack of significant change in the GCS and GCSR in patients who had WMA. The numerical value of GCS and GCSR was noted to be higher in patients without WMA. There was noted to be a significant difference in GCS between rest and stress in patients without WMA and the numerical values were lower at stress in patients who had WMA and perfusion defects. This suggests compromised deformation in patients with evidence of ischemia given by inducible perfusion defect and/or WMA. Our further interpretation is that this gives more objective evidence of altered deformation in patients with perfusion defect and WMA especially when these changes are subtle. Thus, in a patient with borderline perfusion abnormalities, if the strain is depressed, then that would be suggestive of a significant abnormality. This was also reflected in the alteration in the pattern of GCS throughout the cardiac cycle in patients with inducible WMA as shown in Figure 5. Additionally, a systematic review of literature in adult patients with coronary heart disease showed alterations in strain prior to systolic dysfunction (16). All patients in our cohort who have WMA with stress had normal systolic function at the time consistent with the prior finding.

This study is limited by the small, cross-sectional cohort size. The reproducibility of this method across post-processing vendors was not assessed in this study. A larger cohort and longer-term follow-up are needed to determine the difference in these findings across different coronary anomalies and the impact of these findings on clinical and functional status in children with coronary anomalies.

## Conclusions

We conclude that a larger absolute GCS in subjects without WMA or inducible perfusion defect and the lack of significant change in the GCS at peak stress in subjects with inducible WMA are suggestive of compromised LV deformation in subjects with WMA and inducible perfusion defect. Given these findings, GCS derived from CMR-FT may be considered to objectively assess WMA in pediatric patients undergoing DS CMR, especially in patients with different coronary anomalies, and may contribute to risk stratification and clinical decision-making.

## Data Availability

The raw data supporting the conclusions of this article will be made available by the authors, without undue reservation.

## References

[1] Cheezum MK Liberthson RR Shah NR Villines TC O’Gara PT Landzberg MJ Anomalous aortic origin of a coronary artery from the inappropriate sinus of Valsalva. J Am Coll Cardiol. (2017) 69(12):1592–608. 10.1016/j.jacc.2017.01.031 28335843

[2] Davis JA Cecchin F Jones TK Portman MA. Major coronary artery anomalies in a pediatric population: incidence and clinical importance. J Am Coll Cardiol. (2001) 37(2):593–7. 10.1016/S0735-1097(00)01136-0 11216984

[3] Angelini P. Coronary artery anomalies: an entity in search of an identity. Circulation. (2007) 115(10):1296–305. 10.1161/CIRCULATIONAHA.106.618082 17353457

[4] Bianco F Colaneri M Bucciarelli V Surace FC Iezzi FV Primavera M Echocardiographic screening for the anomalous aortic origin of coronary arteries. Open Heart. (2021) 8(1):e001495. 10.1136/openhrt-2020-001495 33431619 PMC7802674

[5] Maron BJ Doerer JJ Haas TS Tierney DM Mueller FO. Sudden deaths in young competitive athletes. Circulation. (2009) 119(8):1085–92. 10.1161/CIRCULATIONAHA.108.804617 19221222

[6] Doan TT Molossi S Sachdeva S Wilkinson JC Loar RW Weigand JD Dobutamine stress cardiac MRI is safe and feasible in pediatric patients with anomalous aortic origin of a coronary artery (AAOCA). Int J Cardiol. (2021) 334:42–8. 10.1016/j.ijcard.2021.04.031 33892043

[7] Nagel E. Moving toward the optimal test for the assessment of myocardial ischemia. J Am Heart Assoc. (2016) 5(8):e003266. 10.1161/JAHA.116.003266 27543304 PMC5015274

[8] Kamal YA Al-Elwany SE Ghoneim AM El-Minshawy AM. Wall motion score index predicts mortality after coronary artery bypass grafting in patients with viable non-functioning myocardium. Ann Cardiovasc Thorac Surg. (2018) 1(2):39–40. 10.35841/cardiovascular-surgery.1.2.39-40

[9] Ingul CB Rozis E Slordahl SA Marwick TH. Incremental value of strain rate imaging to wall motion analysis for prediction of outcome in patients undergoing dobutamine stress echocardiography. Circulation. (2007) 115(10):1252–9. 10.1161/CIRCULATIONAHA.106.640334 17325245

[10] Scatteia A Baritussio A Bucciarelli-Ducci C. Strain imaging using cardiac magnetic resonance. Heart Fail Rev. (2017) 22(4):465–76. 10.1007/s10741-017-9621-8 28620745 PMC5487809

[11] Schuster A Kutty S Padiyath A Parish V Gribben P Danford DA Cardiovascular magnetic resonance myocardial feature tracking detects quantitative wall motion during dobutamine stress. J Cardiovasc Magn Reson. (2011) 13(1):58. 10.1186/1532-429X-13-58 21992220 PMC3217847

[12] Muser D Castro SA Santangeli P Nucifora G. Clinical applications of feature-tracking cardiac magnetic resonance imaging. World J Cardiol. (2018) 10(11):210–21. 10.4330/wjc.v10.i11.210 30510638 PMC6259029

[13] De Silva K Bandara A Schuster A Lamata P Jogiya R Hussain ST Cardiovascular magnetic resonance myocardial feature tracking predicts severity of wall motion abnormalities following acute coronary syndromes. J Cardiovasc Magn Reson. (2013) 15(Suppl 1):P200. 10.1186/1532-429X-15-S1-P200

[14] Meyer SL Ridderbos FJS Wolff D Eshuis G van Melle JP Ebels T Serial cardiovascular magnetic resonance feature tracking indicates early worsening of cardiac function in Fontan patients. Int J Cardiol. (2020) 303:23–9. 10.1016/j.ijcard.2019.12.041 31918854

[15] Bogarapu S Puchalski MD Everitt MD Williams RV Weng HY Menon SC. Novel cardiac magnetic resonance feature tracking (CMR-FT) analysis for detection of myocardial fibrosis in pediatric hypertrophic cardiomyopathy. Pediatr Cardiol. (2016) 37(4):663–73. 10.1007/s00246-015-1329-8 26833321

[16] Mangion K McComb C Auger DA Epstein FH Berry C. Magnetic resonance imaging of myocardial strain after acute ST-segment-elevation myocardial infarction: a systematic review. Circ Cardiovasc Imaging. (2017) 10(8):e006498. 10.1161/CIRCIMAGING.117.006498 28733364

[17] Doctor P Sharma B Greil G Dillenbeck J Abdulkarim M Jaquiss R Dobutamine stress cardiovascular magnetic resonance derived 2-dimension feature tracking strain analysis in pediatric population with anomalous aortic origin of right coronary artery. Pediatr Cardiol. (2024) 45(3):520–8. 10.1007/00246-023-03401-9 38233665

[18] Voges I Negwer I Caliebe A Boroni Grazioli S Daubeney PEF Uebing A Myocardial deformation in the pediatric age group: normal values for strain and strain rate using 2D magnetic resonance feature tracking. J Magn Reson Imaging. (2022) 56(5):1382–92. 10.1002/jmri.28073 35072310

[19] Gobel FL Norstrom LA Nelson RR Jorgensen CR Wang Y. The rate-pressure product as an index of myocardial oxygen consumption during exercise in patients with angina pectoris. Circulation. (1978) 57(3):549–56. 10.1161/01.CIR.57.3.549 624164

[20] Cerqueira MD Weissman NJ Dilsizian V Jacobs AK Kaul S Laskey WK Standardized myocardial segmentation and nomenclature for tomographic imaging of the heart. A statement for healthcare professionals from the Cardiac Imaging Committee of the Council on Clinical Cardiology of the American Heart Association. Circulation. (2002) 105(4):539–42. 10.1161/hc0402.102975 11815441

[21] Klein P Holman ER Versteegh MIM Boersma E Verwey HF Bax JJ Wall motion score index predicts mortality and functional result after surgical ventricular restoration for advanced ischemic heart failure. Eur J Cardiothorac Surg. (2009) 35(5):847–53. 10.1016/j.ejcts.2008.12.046 19272788

[22] Lang SM Frazier EA. Collins 2nd RT. Aortic complications following pediatric heart transplantation: a case series and review. Ann Pediatr Cardiol. (2016) 9(1):42–5. 10.4103/0974-2069.171354 27011691 PMC4782467

[23] Lebeau R Serri K Di Lorenzo M Sauvé C Van Le HV Soulières V Assessment of LVEF using a new 16-segment wall motion score in echocardiography. Echo Res Pract. (2018) 5(2):63–9. 10.1530/ERP-18-0006 29628446 PMC5887066

[24] Savage ML Hay K Anderson B Scalia G Burstow D Murdoch D The prognostic value of echocardiographic wall motion score index in ST-segment elevation myocardial infarction. Crit Care Res Pract. (2022) 2022:8343785. 10.1155/2022/8343785 36405398 PMC9671736

[25] Eek C Grenne B Brunvand H Aakhus S Endresen K Hol PK Strain echocardiography and wall motion score Index predicts final infarct size in patients with non–ST-segment–elevation myocardial infarction. Circ Cardiovasc Imaging. (2010) 3(2):187–94. 10.1161/CIRCIMAGING.109.910521 20075142

[26] Pedrizzetti G Claus P Kilner PJ Nagel E. Principles of cardiovascular magnetic resonance feature tracking and echocardiographic speckle tracking for informed clinical use. J Cardiovasc Magn Reson. (2016) 18(1):51. 10.1186/s12968-016-0269-7 27561421 PMC5000424

[27] Molossi S Sachdeva S. Anomalous coronary arteries: what is known and what still remains to be learned? Curr Opin Cardiol. (2020) 35(1):42–51. 10.1097/HCO.0000000000000696 31633566

